# Influence of Carbyne Content on the Mechanical Performance of Nanothick Amorphous Carbon Coatings

**DOI:** 10.3390/nano10040780

**Published:** 2020-04-18

**Authors:** Ana P. Piedade, Liliana Cangueiro

**Affiliations:** 1 Department of Mechanical Engineering, CEMMPRE, University of Coimbra, Rua Luis Reis Santos, 3030-788 Coimbra, Portugal; 2 CeFEMA-Instituto Superior Técnico, University of Lisbon, Av. Rovisco Pais 1, 1049-001 Lisboa, Portugal; lilianacangueiro@gmail.com

**Keywords:** 2D carbon thin films, sp^1^ carbon bonds, nanocrystalline copper coating, zero coefficient of friction

## Abstract

This study concerns the evaluation of the coefficient of friction, at different temperatures, of amorphous carbon thin films, deposited onto nanocrystalline sputtered copper coatings by clean-technology rf magnetron sputtering. The aim is to access the capacity of carbon thin films, with different contents of sp^2^ and sp^1^ bonds, to act as a solid lubricant for copper surfaces. Raman spectroscopy revealed that all the as-deposited coatings consist of amorphous carbon with low defect content and decreasing carbyne concentration with increasing thickness. The tribological tests at 25 °C and 200 °C revealed that, for the higher temperature, the 15 nm carbon coating present 0.001 friction coefficients at 2 N load. Overall, the study presents a one-step technology for the greener production of solid lubrication systems for micro- and nano-components, avoiding the environmental impact of lubricants.

## 1. Introduction

Pure metals thin films, such as copper, are already being used and are also emerging as potential candidates to replace Si in specific micro and nano-electromechanical systems (MEMS/NEMS) [1,2]. Due to circuitry miniaturization, the distance between layers decreases in size, and the conventional process of electroplating for the manufacture of interconnects becomes increasingly more inefficient, due to the presence of unfilled gaps within the structure [3,4]. The limitations of this metallization process have raised the hypothesis of replacing the entire electroplating process with sputtering deposition technology [5]. Additionally, it must also be considered that, at temperatures around 200 °C, copper forms a relatively dense oxide, with thickness ranging from tens to a few hundred nanometers, and thicker for higher temperatures [6]. Considering that channel gaps in MEMS devices are usually in the range of 100 to 1000 nm, lower for NEMS, the formation of the metallic oxide strongly interferes with dimensional accuracy and, consequently, with the performance of the component [7]. The copper components in these devices are subject to occasional tribological contact with other parts. Therefore, both oxide formation and wear results in severe damage to the components. Moreover, under these micro and nanometric confined geometries, liquid base lubricants may not be considered due to many constraints, including surface tension issues [7]. All these limitations impose severe problems in the search for valid solid lubricants to be used in the mentioned applications.

Graphene has risen during the last decade as a super carbon due to the unique set of properties resulting from its two-dimensional arrangement [8]. Among these properties, its use as lubricant has been reported, however mostly as an additive to water and other liquid-based lubricants and never as a protective coating for copper substrates [9,10]. Nonetheless, graphene and graphene-based materials (GBM), such as graphene oxide (GO) or reduced graphene oxide (rGO), have been described as solid lubricants for MEMS/NEMS applications, with other materials [11,12,13]. However, researchers highlighted the fact that there are several drawbacks in the use of these systems, including the defects of the top layer of graphene and GBM, which strongly influences the tribological behavior of these materials [14,15]. Additionally, the production of graphene usually implies a transfer step of the carbon layer from the support material, which by itself can condition the quality of the graphene [16], to the substrate where it is intended to be used. This procedure implies that defects, such as wrinkles and fissures, usually appear and seriously compromise the exceptional results expected from these materials [17]. 

Moreover, the outstanding properties attributed to graphene refer to the carbon allotrope in the free-standing mode, as the presence of a substrate always induced the impairment of such properties [18]. Also, many of the experimental techniques used for the production of these carbon allotropes use high temperatures, such as chemical vapor deposition, which makes them incompatible for the deposition in several metallic substrates, including copper. Similarly, when considering the reduction of GO, the chemical treatments required for obtaining rGO are concomitantly responsible for introducing chemical defects and impurities on the layers of graphene, thus contributing to degradation of the overall performance [19]. 

The tribological properties of carbon allotropes are the subject of a considerable number of published work, such as the nano- and micro-tribology of graphene and GBM [20,21,22], but very few that report the study of the friction behavior of these materials under macro loads. Among these, a study reported the five-fold reduction of the friction coefficient between a steel ball and a steel disk when a single layer of graphene was used between the sliding surfaces, but only in a hydrogen atmosphere [23]. The presence of hydrogen was mandatory for the improvement of the friction coefficient as it acts as a bond element for the carbon dangling bonds that occur when damage or rupture of graphene carbon bonds occurs. However, this particular atmosphere does not mimic the actual conditions that copper coated components experience in MEMS/NEMS devices. For these reasons, some other methods are now being considered for the production of carbon allotropes, other than graphene, to evaluate their applicability. Among the techniques for the production of coatings, sputtering is considered a very versatile and green methodology. Specifically, in the area of tribology, the deposition of diamond-like coatings (DLC) [24,25,26] and doped amorphous carbon coatings (d-aC) [27,28,29] are widely reported. However, the properties of DLC, particularly the mechanical ones, are not compatible with softer substrates such as copper. Due to the deposition technique, strains can occur in the growing coating that increase with film thickness resulting, in some cases, in the delamination of the coatings. 

The present work reports the production of copper thin films by radiofrequency (r.f.) magnetron sputtering to act as substrates for carbon thin film deposition. This technique allows for obtaining tailor-made surfaces and structures that are unique and can almost be exclusively obtained by this processing technology [30,31]. Moreover, from previous work [32], it is known that the appropriate choice of deposition parameters induces preferential crystallographic orientations conferring the metallic thin films increased protection against oxidation. Therefore, the deposited copper thin films were coated with nanothick carbon coatings to evaluate their tribological properties under macro loads at different temperatures.

## 2. Experimental Section

### 2.1. Thin Film Deposition

All the depositions were performed using an r.f. sputtering equipment, Edwards Coating System E306A (Edwards, Birmingham, AL, USA), equipped with two power supplies of 1000 and one of 500 W, branched to the two assisted magnetron targets and substrate holder, respectively. Copper target was purchased from Goodfellow, Huntingdon, UK, and graphite target was purchased from Testbourne Ltd., Basingstoke, UK. Both materials were 5 mm thick, 100 mm in diameter, 99.999% purity. All the depositions were carried at 3 × 10^−4^ Pa ultimate vacuum pressure and using argon as the discharge gas. The depositions were all carried out in non-reactive mode at 0.7 Pa total discharge pressure. The variable deposition parameters are summarized in Table 1. For the final depositions, copper and subsequently carbon thin films were deposited onto 100Cr6 (DIN, 5210 AISI) steel (50 mm diameter and 5 mm thickness).

### 2.2. Thin Film Characterization

Morphology and topography: SEM (Scanning Electron Microscopy) was used for surface and cross-section morphological characterization. A FEI Quanta 400FEG ESEM equipment (FEI, Hillsboro, OR, USA) was used. The cross-sections were obtained by sawing the steel substrate at the uncoated part and stopped before reaching the coating; brittle fracture of the substrate/coating was obtained by freezing the samples into liquid nitrogen and applying force onto the uncoated end of the substrate. After cleaning the fractured surface with air current, the coatings were observed and the thickness measured at three different locations from the SEM cross-section micrographs. The topography was observed by AFM (atomic force microscopy) analysis with scans of 1 × 1 μm^2^. A Veeco, diInnova equipment in tapping mode using Si_3_N_4_ RTESPA-CP tips, from Bruker, with a resonance frequency of 291–326 kHz and spring constant of 20–80 N·m^−1^.

Microstructure: XRD (X-ray diffraction) was performed in a Phillips X’Pert diffractometer (Panalytical, Kassel, Germany) equipped with a PW3200/00 goniometer and a cobalt anticathode (λK_α1_ = 0.178896 nm). The diffractograms were obtained with a tension of 40 kV and a current of 35 mA. TEM (transmission electron microscopy) studies were performed on a Tecnai G2 microscope. The thin films were deposited directly onto an appropriated TEM grid. 

Chemical: The XPS (X-ray photoelectron spectroscopy) analyses were performed in a VG–ESCLAB 250iXL spectrometer (Kratos Analytical, Manchester, UK), as described earlier [33]. Briefly, the pressure in the analysis chamber was kept below 5×10^−8^ Pa, and the analysis was performed using monochromatic radiation Al-Kα (h*ν* = 1486.92 eV). The photoelectrons were collected with an angle of 90° regarding the surface of samples. The energy step was of 20 eV for the survey spectra and of 0.05 eV for the high-resolution spectra. A flood type charge neutralizer attached to the spectrometer was used simultaneously during the measurement. Calibration of binding energy was carried out using the spectrum of Ag 3d5/2 (368.3 eV) before and after sample measurements. The chemical compositions were obtained using the sensitivity factor of the Scofield library. The deconvolution of the high resolution spectra was made through CasaXPS^®^ Software (version 2.3.20, Casa Software, Lda) using Voigt function. The surfaces were also characterized by micro-Raman spectroscopy, as well as the wear tracks produced during the friction tests for the tribological characterization. A Horiba LabRAM HR800 Evolution (Jobin-Yvon, Montpelier, France) spectrometer equipped with an Olympus BXFM confocal microscope was used, with a solid-state laser operating at 532 nm and a power of 0.25 mW reaching the sample. The spectra were obtained with an acquisition time of 3 s and 10 accumulations. The system was calibrated to better than 1 cm^−1^ before the measurements, using a silicon sample. A spectrograph with a 600 lines/mm grating was used to provide a spectral resolution of 2 cm^−1^, and the confocal hole size was set at 150 µm. 

Contact angle measurements: The wettability characteristics of thin films were assessed by measuring the static contact angle of the surfaces with 10 μL of distilled and deionized water, in a DataPhysics QCA-20 143 apparatus (DataPhysics, Filderstadt, Germany). For each sample, triplicates were used, and a minimum of seven measurements was taken after allowing the system (air–water–surface) to reach equilibrium, and the average value calculated. 

Mechanical properties: Hardness (*H*) and Young’s modulus (*E*) were evaluated by nanoindentation depth-sensing indentation (MicroMaterials, Nano Tester, Camarillo, CA, USA) using a Berkovich diamond indenter. In all surfaces, the load was set to 0.5 mN with 30s for the loading and 30s for the unloading with 5s maintenance at maximal load. Twenty indentation measurements were recorded on each specimen. The coefficient of friction was evaluated in a conventional ball-on-disk homemade equipment. Both temperature and humidity were controlled, and a charge cell Sensotec was used for the acquisition of the coefficient of friction by the computerized control system, with a friction coefficient error of 0.001 at 2 N load. The balls, with 10 mm diameter, were of 316L (AISI) stainless steel while the disks (50 mm diameter) used as substrates were of 52100 (AISI) steel. The disks were coated with Cu and with Cu-C thin films, and for comparison purposes, bulk copper was also tested. The tests were performed at 30% of relative humidity, temperatures of 25, 100, and 200 °C, with loads of 1 N, 2 N, and 5 N, for 500 cycles. Each test was repeated at least three times for reproducibility assessment.

## 3. Results and Discussion

### 3.1. Characterization of the Thin Films

**Figure 1:**
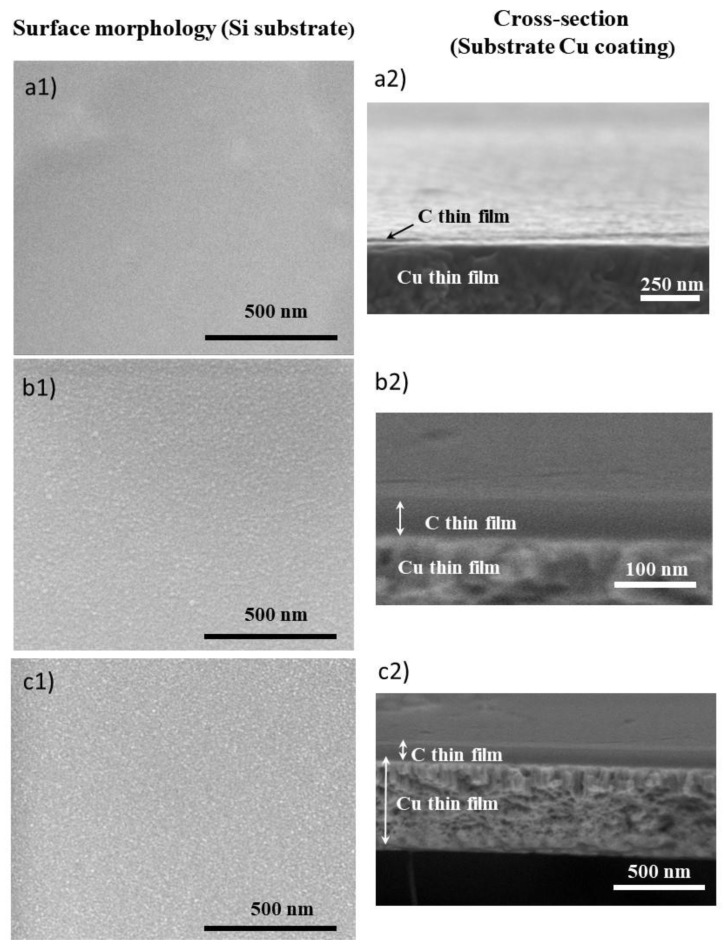
Scanning electron microscopy (SEM) micrographs of C thin films (**a**) C15, (**b**) C60, (**c**) C100. Surface morphology onto Si substrate and cross-section onto Cu thin film.

**Figure 2:**
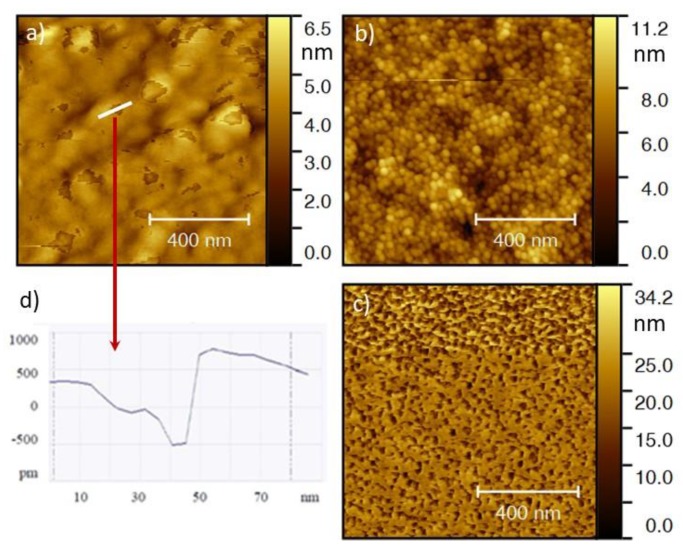
Atomic force microscopy (AFM) surface topographic images of (**a**) C15, (**b**) C60 and (**c**) C100 thin films deposited onto Cu coating. (**d**) 2D profile of the line highlighted in image (**a**).

Surface and cross-section morphologies of copper and carbon deposited thin films were characterized by SEM and are depicted in Figure 1, and the corresponding deposition conditions, designation, and thickness are summarized in Table 1. Considering that the morphology can be influenced by the substrate, the carbon thin films were characterized when deposited directly onto the silicon substrate as well as on the surface of the copper thin film (Cu). The cross-section morphology revealed that all carbon thin films are compact, dense, porous free, and featureless regardless of the substrate on to which they are deposited. On the contrary, surface topography accessed by AFM revealed quite different topographies for the films deposited onto Si or Cu (Figure 2), as expected. The surface topography of Si-deposited films presents the ‘true’ carbon coatings morphology, whereas the deposition onto the copper thin films is conditioned by the topography of the top surface of the metallic coating. 

For the thinner carbon coating deposited onto Cu thin film (Cu-C15), it is possible to see some surface features that could be considered defects. However, a 2D profile over these regions (Figure 2d) shown that they correspond to areas where the out-most layer of the thin film has not able to form a continuous layer. The depressions present a thickness of around 1 nm. These observations indicate that the thinner carbon thin film is deposited by layers and not as ‘islands’ or agglomerates of atoms, which are two of the possible mode of thin film growth. The layered disposition of the carbon coating indicates that upon deposition, the cohesive energy was higher than the adhesive energy [34,35], which is normal given the lack of chemical affinity between copper and carbon. This is corroborated by the lack of publications concerning the deposition of C thin films on Cu in the literature and is one of the reasons with cooper, along with nickel, is used as “sacrificial” substrate for the growth of carbon allotropes [36].

However, as the carbon thin film thickness increases the difference between cohesive and adhesive energies is mitigated because the bottom layers are now of the same material that is being deposited carbon. Therefore, the morphology and topography are now ruled by the minimization of surface energy which is responsible for the changes of the topography as the thickness increases.

The chemical characterization by XPS revealed only the presence of carbon and oxygen. The content of the latest was approximately 10 at% in all the deposited coatings. The presence of oxygen is not surprising as the films present defects as already detected in the previous AFM analysis. Besides the normal defects on the edges of each layer, in the case of C15 thin film more defects appear resulting from a non-continuous layer of atoms at the out-most surface. Moreover, during the deposition, the presence of residual oxygen in the sputtering chamber will induce a preferential reaction of this element with the dangling C bonds. Thermodynamically, it is always more favorable the formation of carbon-oxygen bonds that carbon-carbon bonds (Δ*H_f_* in kJ·mol^−1^ is −346 for C–C; −602 for C=C; −358 for C–O; and −799 for C=O) [37].

**Figure 3:**
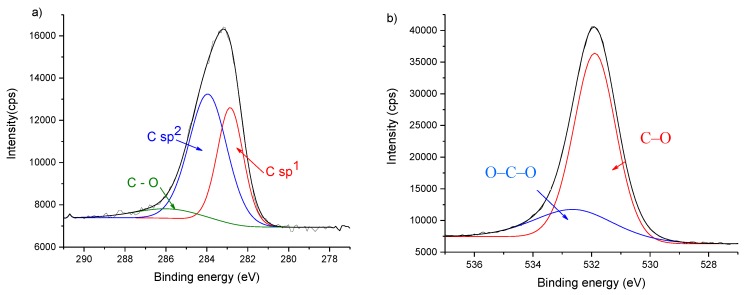
Deconvolution of the high resolution spectra of surface Cu-C15: (**a**) C1s, indicating only the presence of carbyne (sp^1^) and graphene-like (sp^2^) hybridized bonds, as well as the presence of C–O bonds; (**b**) O1s, showing that oxygen is manly bounded to carbon.

Figure 3 displays the deconvolution of the high resolution XPS spectra of the Cu-C15 thin film for C 1s and O 1s. The results of the deconvolution from XPS data for all the C thin films, including full-width at half-maximum (FWHM), are summarized in Table 2.

The obtained results show that the amount of sp^1^ carbon hybridized bonds (282,6 eV [38]), attributed to carbyne bonds decreases (from 36% to 0%) with the increase of thickness, in opposition with C sp^3^ bonds that increase from Cu-C15 (0%) to Cu-C100 (34%). The interesting result from this characterization is the absence of sp^3^ carbon hybridized bonds in the C15 surface, while sp^1^ bonds represent around 36% of the total of the carbon.

**Figure 4:**
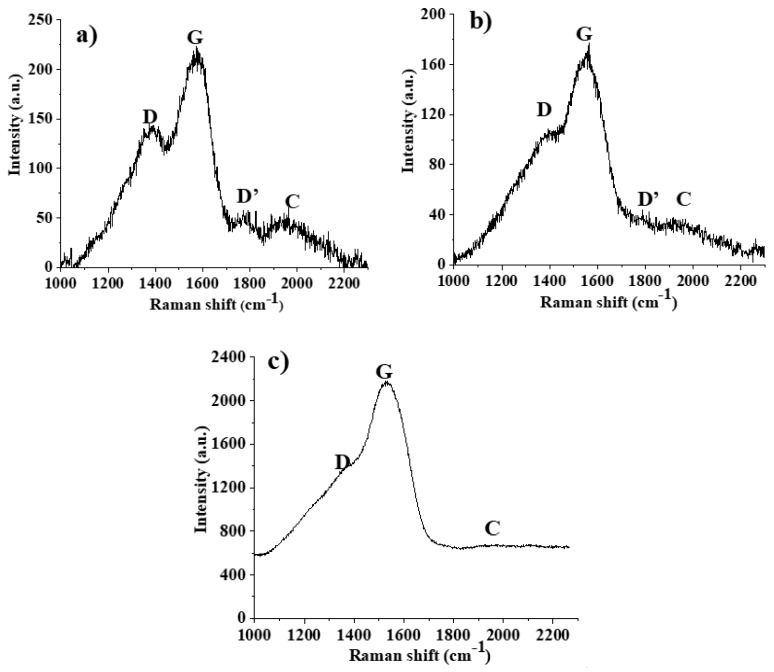
Micro-raman spectra of carbon thin films: (**a**) C15, (**b**) C60, and (**c**) C100.

However, it must be highlighted that it is only the top few surface layers of each coating are analyzed. Therefore, any study of the sp*^x^* ratios and discussion relating these values using XPS data will represent only the surface values, and not necessarily those of the entire volume of the thin film. This observation implies that the results for Cu-C15 thin film correspond to almost all the thickness of the coating, whereas for Cu-C100 the analysis corresponds to about 1/10 of the coating thickness. Considering that the tribological tests, due to the experimental conditions especially the macro load, will be influenced by the total amount of the carbon thin films, additional characterization was performed. Therefore, the entire volume of the three carbon thin films was evaluated by micro-Raman spectroscopy (Figure 4).

The spectra profile between 1200–1700 cm^−1^ is similar to amorphous carbon and is related to vibrational modes of sp^2^-hybridized locations [39]. For C15 and C60 thin films it is more noticeable a peak 1760 cm^−1^ (the D’ peak) [40], and a broad band between 1800–2250 cm^−1^ (marked as C) which is attributed to carbyne peak [41]. These are linear carbon chains with sp^1^ hybridization that can be polyynes of alternating triple and single bonds (C–C≡C)_n_, and polycumulenes as linear double bonds carbon chains (C=C)_n_ [41]. The D and D’ bands also appear in Raman spectra although they are Raman-forbidden, according to the Raman selection rule [40]. The deconvolution of the spectra allowed to quantify the relative intensity ratios of the identified peaks, as well as the position of the G peak (Table 3). 

The results show that a more disordered structure emerges with increasing thickness, as evaluated by the downshift of the G peak position, as compared with that of pure graphene which occurs at 1580 cm^−1^ [42]. The more ordered structure corresponds to the thinner carbon film as it is the most similar to a graphene multilayer arrangement, while the thicker carbon coating is closer to a graphitic material. These observations are in accordance with the contact angle measurements with water that increases its value with increasing thickness of the carbon thin films: 52 ± 2°, 63 ± 1°, and 78 ± 1° for C15, C60, and C100, respectively. This trend is also in agreement with other works where it has been reported that a single graphene layer onto a glass surface presents a contact angle of 48° and this value increases with the number of layers until it reaches the value for the graphite surface which is of 92° [43].

Usually, the literature uses the ratio of the intensity of the D peak, to the G peak (*I_D_/I_G_*), to estimate the defects in graphene and other carbon allotropes, attributing it to sp^3^ hybridization related defects [44,45,46]. However, it has been experimentally established that the *I_D_/I_D’_* ratio can be used to get information on the nature of defects in graphene [47]. The authors demonstrated that high values of this intensity ratio (around 13) are related to sp^3^ induced defects. However, moderate values of this intensity ratio, ≈7.5, can be attributed to vacancies defects, while lower values, around 3.5, were correlated with the presence of boundary defects, such as C–O bonds. The same work pointed out that in highly disordered structures the D’ band tends to fuse with the G band being harder to be identified. Following the same reasoning for the carbon thin films, for the thinner ones the values of *I_D_/I_D’_* suggest that the defects are predominately due to boundaries. The C100 thin film—that due to the downshift of the G peak was already considered the less ordered structure—presents a Raman spectrum where it is very difficult to discern, without deconvolution, the presence of the D’ according to the aforementioned work [47]. Also, the *I_D_/I_D’_* for the C100 film indicates that the defects are mainly due to vacancies in the thin film.

**Figure 5:**
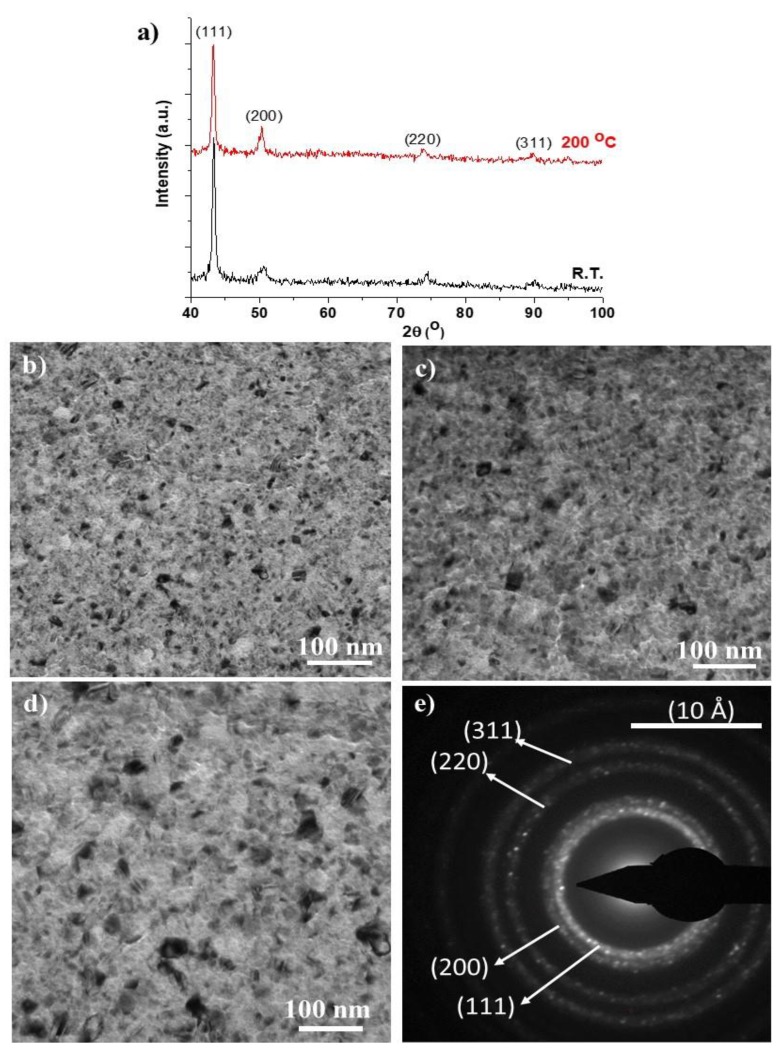
Structural characterization of the Cu thin film: (**a**) X-ray diffractogram, at room temperature and after 24h at 200 °C; (**b**) Transmission electron microscopy (TEM) bright field image of the as-deposited coating; (**c**) TEM bright field image after 2h at 200 °C; (**d**) TEM bright field image after 24h at 200 °C; (**e**) electron diffraction pattern of the area showed in (**a**).

The structural evaluation of the Cu thin film was made by XRD and TEM (Figure 5). In Figure 5a the X-ray diffractograms, at room temperature and 200 °C, show evidence of a preferential orientation according to the (111) diffraction plane. This can be quantified by the ratio between the intensity of the (111) and (200) diffraction planes (I_111_/I_200_) and comparison with the same ratio of the copper ICDD reference card no. 83-1326. The ratio is 2.3 for the reference card, 8.2 in the deposited thin film and 7.8 after 24h at 200 °C. The preferential growth of the (111) diffraction plane is known to occur in certain deposition conditions for materials with faced center cubic (fcc) crystallographic structures [32]. The (111) corresponds to the plane of highest atomic density in the fcc structure, and also the lowest surface energy, thus implicating that the reactivity of the surface is low and that the diffusion of reactive species, such as oxygen, is more difficult. Therefore, the oxidation resistance of the coating material can be significantly higher than the correspondent 3D material or thin films without any preferential crystallographic orientation. Moreover, the width of the diffraction peaks indicates a nanocrystalline material. Characterization through TEM confirmed that the as-deposited coatings (Figure 5b,e) are nanostructured. 

As the Cu coating will play the role of substrate for the depositions of carbon thin films that are going to be subject to tribology tests at different temperatures, the Cu thin film was subjected to thermal treatments at 200 °C in air for 2 h (Figure 5c) and 24 h (Figure 5d) in order to ensure that the results from the tribology tests were not influenced by a structural rearrangement of the metallic coating. None of these procedures changed the nanocrystalline nature of the thin film, and no significant grain growth was observed. 

This result implies that the preferential orientation according to the highest atomic dense crystallographic plane seems to be able to protect the thin film against oxidation and microstructural changes. These can occur for sputtered Cu thin films at temperatures as low as 75 °C [48].

### 3.2. Mechanical Properties

The results from the hardness and elastic modulus evaluated by nanoindentation are summarized in Table 4. For comparison purposes, the result of the hardness of the bulk copper evaluated with 1 N is also displayed.

The results show no significant changes in hardness values of the three deposited carbon thin films, that are also similar to the Cu thin film. These results show that due to the very thin thickness of the C coatings, and although a very small load was used, the values may be influenced by the metallic thin film. It must also be highlighted that the value of the bulk metallic material is higher than those usually reported for copper because a very low load is being applied for comparing the results. This implies that the naturally oxidized surface of copper contributes significantly to the value [49], thus rising it as it can be verified by the hardness determination at higher loads (1 N) which presents a value of hardness half the one determined by nanoindentation. This result is due to the higher volume that is plastically deformed and, consequently, the contribution of the surface oxides diminishes.

The carbon thin films present a decrease in Young’s modulus with increasing thickness of the coatings. This mechanical property is intrinsic depending only on the chemical nature of the material, more specifically the strength of the chemical bonds. All three coatings present the same type of hybridized carbon bonds. However, their relative ratio is different with increasing thickness: the sp^2^ content increases while sp^1^ decreases. Also, the ordered structure decreases and the vacancy defects increase with increasing thickness, as established before. Moreover, and considering that E value of graphite is around 10 GPa [50], it would be expected that the thicker film should present even lower values of Young’s modulus. Considering all this information and the results from the previous characterizations it can be assumed that, with increasing thickness, the observed decrease of E values can be related with the increasing number of defects due to vacancies, as well as with the decreasing percentage of carbynes. The increased number of vacancies implies more disrupted chains, less dense material and therefore when a load is applied a higher strain is induced. Considering the carbyne content, some experimental work, as well as molecular dynamics simulation studies [51,52,53], demonstrated that mechanical properties of linear carbyne chains surpass those of graphene. It is predicted Young’s modulus of 4.6 TPa for carbynes containing more than 10 to 12 atoms of carbon, at T = 0 K, higher than the 1TPa for graphene. Although in small percentages, the presence of carbynes in the thin films developed in this work seems to influence the mechanical properties of the carbon coatings, with special emphasis in the elastic modulus that can counteract the effect of the vacancies and the graphitic-like structure of the thicker thin film.

**Figure 6:**
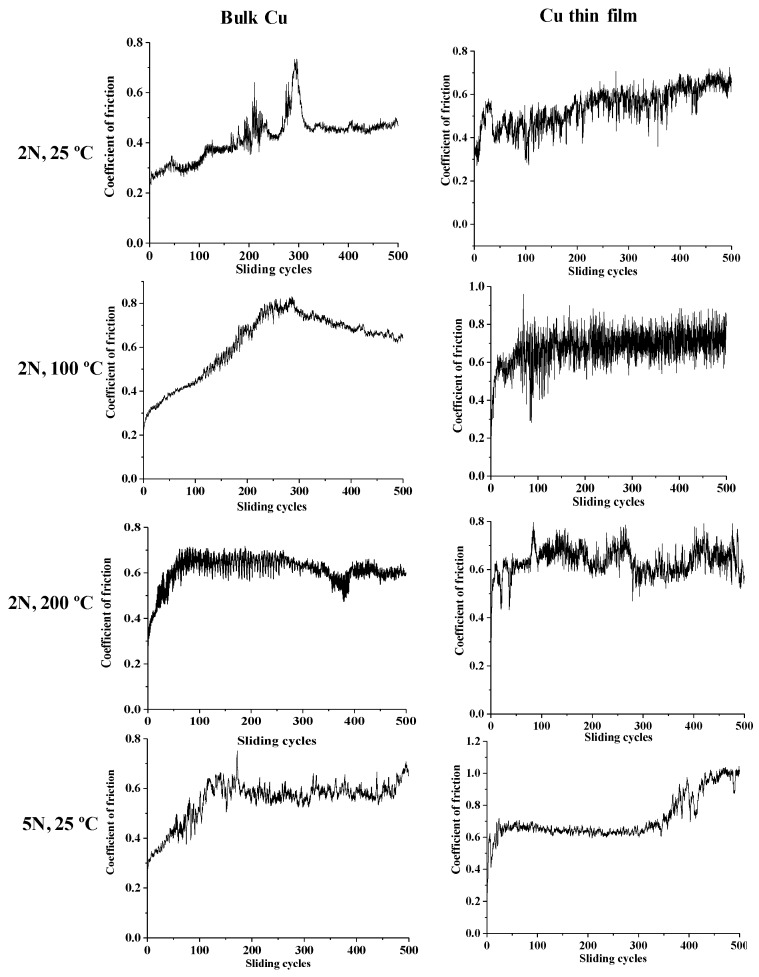
Coefficient of friction of bulk copper and Cu thin film at different loads and different temperatures.

In what concerns the tribological properties, the coefficient of friction of copper, in bulk and as thin film were measured at different temperatures and loads for reference purposes. The results are present in Figure 6.

These experiments showed that at loads of 5 N the copper thin film was removed from the substrate after around 300 sliding cycles, and the behavior of the cooper, in bulk and as thin film, is similar. In fact, due to the low number of sliding cycles, it is not possible to perceive the oxidation protection of the surface that is expected from the Cu thin film, as shown by the TEM characterization after the thermal treatments. 

**Figure 7:**
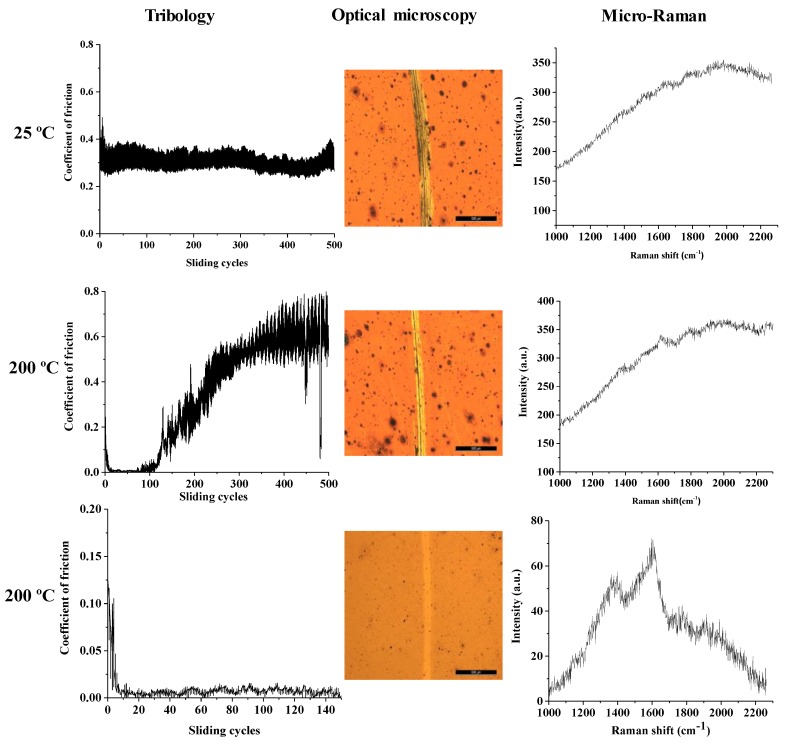
Coefficient of friction, optical macrographs, and micro-Raman characterization of the wear tracks of Cu-C15 after tribology tests, with 2 N load at different temperatures (bar = 500 μm).

**Figure 8:**
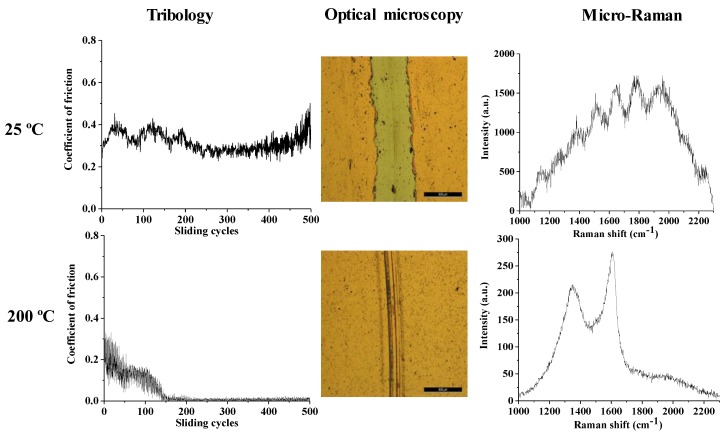
Coefficient of friction, optical macrographs, and micro-Raman characterization of the wear tracks of Cu-C60 after 500 sliding cycles, with 2 N load, at different temperatures (bar = 500 μm).

**Figure 9:**
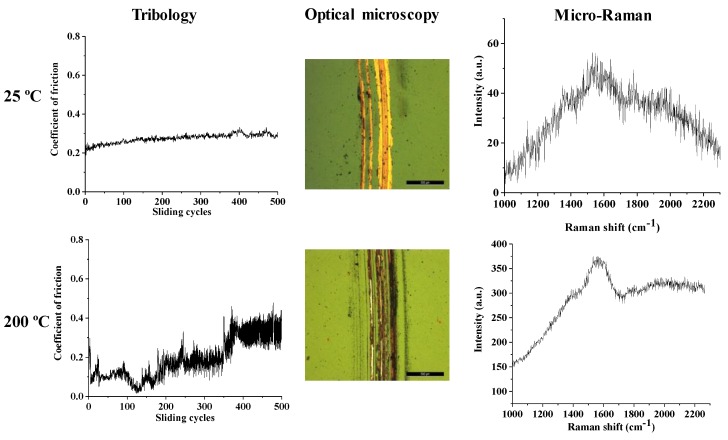
Coefficient of friction, optical macrographs, and micro-Raman characterization of the wear tracks of Cu-C100 after the tribology tests, with 2 N load at different temperatures (bar = 500 μm).

Since the results of this tests demonstrated that the Cu film is removed if a load of 5 N is used, and after repeating the tests of the Cu thin films at 2 N load, the friction tests on Cu coated with carbon thin films were carried out only with a low of 2 N at room temperature (25 °C) and for the higher temperature, 200 °C. Besides the evolution of the coefficient of friction with the number of sliding cycles, each of the produced wear tracks was analyzed by optical microscopy and micro-Raman spectroscopy. The results for the Cu-C15, Cu-C60, and Cu-C100 surfaces are present in Figure 7, Figure 8 and Figure 9, respectively. The results will be presented for the three systems and an integrated discussion will follow.

For the Cu-C15 (Figure 7) at room temperature, it is noticeable that the presence of the carbon thin film induces a coefficient of friction (μ = 0.3) smaller than the uncoated Cu thin film (μ = 0.5–0.6). However, the coefficient of friction begins to increase after the 500 sliding cycles due to the removal of the C coating, as confirmed by the “abnormal” micro-Raman spectrum, as there is no Raman signal on metals. If the number of sliding cycles was higher, it would be expected that the coefficient of friction would rise to that of the Cu thin film.

The behavior of this film at high temperature is completely different. The presence of the carbon coating induces an extremely low coefficient of friction for the first 150 cycles and then the value rises to attain the one of the copper thin film. Once again, this increase is due to the removal of the carbon coating as confirmed by micro-Raman spectroscopy. The tribology tests were then repeated but only for 150 sliding cycles and, similarly to the previous experiments, the coefficient of friction was extremely low. The wear track shows a very different aspect as almost no wear is detected and the micro-Raman analysis confirm that the carbon coating is still present in the wear track. The same technique also highlights the fact that the thin film maintains the carbyne hybridized bonds and that a more ordered structure was formed, as showed by the beginning of the separation between the D and G peaks. 

Very similar behavior was observed for the Cu-C60 system (Figure 8). At room temperature, the coefficient of friction is around 0.3 with a tendency to increase this value after 500 sliding cycles, as observed before. This behavior is also due to the removal of the carbon coating during the application of the macro load, as observed in the micrograph and confirmed by the corresponding micro-Raman spectrum. Once again, the tribological properties of the coatings at higher temperature are different. The initial value for the coefficient of friction is around 0.3 and decreases during the first 150 sliding cycles until it reaches an extremely low friction coefficient, that is maintained until the end of the test at 500 sliding cycles. The micro-Raman analysis reveals that the C thin film is still present on the surface of the copper coating and it is characterized by the presence of both the carbyne peak and the separation between G and D peaks, as observed for the thinner carbon coating.

Moreover, an additional experiment was made in this system: after 300 sliding cycles the test was stopped and the temperature allowed to drop until room temperature. The test was restarted at room temperature and the extremely low coefficient of friction was maintained until de 450 (total) sliding cycles and then started to rise very slowly to a value around 0.05 after the 500 sliding cycles. This supplementary result further reinforces the idea that the changes in the microstructure of the sliding interface change are responsible for attaining the extremely low values of the coefficient of friction.

The study of the Cu-C100 system (Figure 9) shows a more distinct behavior than the one observed for the thinner carbon thin films. At the end of the tests, the carbon thin film is present in the wear track, although much more visible after the test at 200 °C than at room temperature. At room temperature, the coefficient of friction is relatively constant and around 0.3, without the increasing tendency, observed in the previous coatings, although with a less sharp profile. This may be a consequence of the higher thickness of the carbon coating although it is noticeable by the optical micrograph that the majority of the coating has been removed after the 500 cycles. At higher temperatures, the coefficient of friction decreases during the first 150 cycles, similarly to what occurs in Cu-C60, but it does not remain around 0.001 as for Cu-C60 coating. Instead, it starts to rise, although from the optical observation and from the Raman spectra it is clear that the carbon coating is still present.

**Figure 10:**
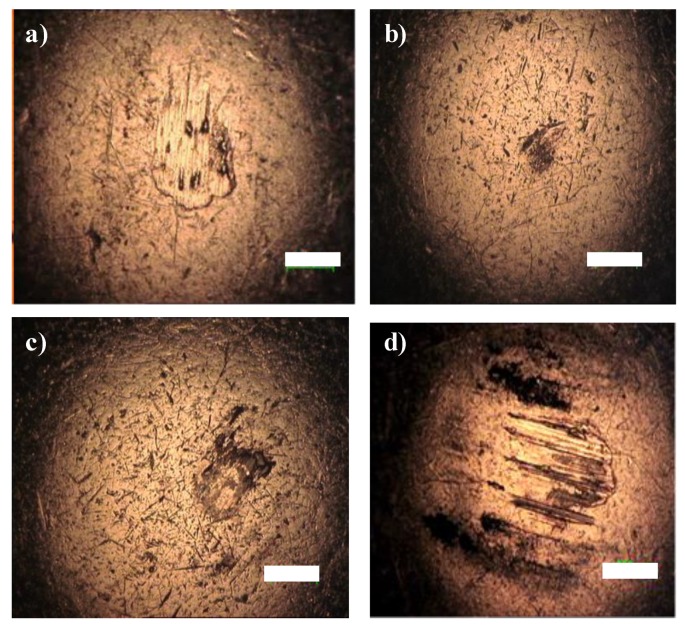
Optical macrographs of the steel balls after the tribology tests, at 200 °C, with the carbon coatings: (**a**) Cu-C15, 500 cycles; (**b**) Cu-C15, 150 cycles; (**c**) Cu-C60, 500 cycles; (**d**) Cu-C100, 500 cycles (bar = 200 μm).

Also, from the micrographs of the wear tracks it is possible to conclude that with increasing thickness of the coating, the wear tracks (after 500 sliding cycles) is wider. This observation was confirmed by the optical micrographs of the steel ball used in each test (Figure 10).

The analysis of the steel surfaces shows that with increasing thickness of the coatings, larger areas of carbon are transferred to the steel counter-body.

Relevant information is available on the nano- and micro-scale tribology of carbon-based materials. Regarding the macro-scale tribology of these materials, very few are the studies that report results from tribology where loads of 1 N or higher are used. As previously stated, usually, these concern studies were the carbon allotrope is added to a lubricant solution or a liquid suspension [9,10]. Considering that this study aims at evaluating the ability of carbon surfaces to act as solid lubricants, these results obtained by other authors cannot be considered for discussion purposes.

The analysis of the results of the tribology tests of this work at room temperature seems to indicate they are mainly governed by the thickness of the carbon coating. All carbon thin films are deposited onto the same substrate, present the same hardness, and the steel balls used in the tests do not have a polished surface and present many defects. Although the coefficient of friction is relatively similar and around 0.3 for all the deposited thin films, the stability of the value through the entire test is much higher for the thicker coating, decreasing with decreasing thickness. As soon as the steel ball removes all the carbon coating, the coefficient of friction begins to increase. As reported, the Cu-C100 system presents properties similar to graphite, and it is long known that under humid conditions, such as the ones of the tests of this work at room temperature, graphite presents better tribological properties due to intercalation of water molecules between the graphite layers [54]. Consequently, better detachment of individual layers occurs that reduces the friction in the sliding interface. For the Cu-15 and Cu-C60 coatings that are not graphitic-like, the presence of moisture can induce stronger adhesion between carbon layers in the form of hydrogen bonds, which gives these coatings the necessary resistance to endure the wear by macroloads. However, the low thickness of the carbon thin film can only induce moderate protection, as due to the 2 N applied load, these coatings are quickly removed from the copper surface. 

For higher temperatures, the carbon coatings show entirely different behavior. The type of carbon bond, the degree of structural order/disorder—together with the mechanical properties, especially the elastic modulus—seems to be responsible for the observed results. The Cu-C15 thin film attains an extremely low coefficient of friction almost immediately after the beginning of the test, and this value is maintained for the first 150 sliding cycles. After this number of cycles, the coating begins to be removed, and the coefficient of friction increases. This surface is the most similar to multilayer graphene, presents the highest Young’s modulus, a higher percentage of carbynes, and the defects are mostly due to boundaries. From the results, it is also visible that the first 150 sliding cycles induce a higher degree of structural order. This behavior may also be a consequence of the lack of extra-hydrogen bonds between layers due to the absence, at a higher temperature, of residual moisture. Without the hydrogen bonds, the detachment of individual layer of the coating for the formation of a tribofilm between the coating and the steel ball during these first cycles is enhanced, thus lowering the coefficient of friction. Moreover, the facility to break the remaining van der Walls secondary forces allows for the metastable thin film to suffer a microstructure evolution, according to the micro Raman spectra after 150 cycles at 200 °C (Figure 7). Also, the higher Young’s modulus of the three deposited coatings implies that smaller strain will be experienced by the film, which suggests a smaller wear track without the smearing of the interface tribofilm, which is confined to the wear track.

When considering the results of Cu-C60, it seems that the previous explanations also apply, except for the fact that the first 150 sliding cycles correspond to an adaptive stage. This stage corresponds to the thinning of the coating by transferring material to the steel ball, concomitantly with the appearance of a more ordered structure that it can be considered more similar to multilayer graphene rather than a graphite material. Due to the lower elastic modulus of this coating when compared with Cu-C15, the Cu-C60 coating suffers higher strain in the same load conditions, thus inducing a higher contact area with the steel ball giving rise to a broader wear track. After this first stage, the test reaches an extremely low coefficient of friction that is maintained during the rest of the sliding cycles. The longevity of these extremely low values is due to a thicker coating that during the first cycles is being transferred to the steel ball resulting in a multilayer graphene type carbon coating both on the surface of the copper thin film and on the steel ball. Due to the applied load, the carbon on the copper surface presents a more ordered structure, and a sliding interface between transferred carbon tribofilm in the steel ball and the Cu-C60 coating is observed. These two facts highly contribute to attaining and maintaining an extremely low coefficient of friction, as already discussed for the Cu-C15 case. 

The Cu-C100 coating presents a similar behavior to Cu-C60 surface for the first 150 sliding cycles. The reason must also be related to the fact that during this phase, the transfer of material from the coating to the steel ball is occurring with the formation of a very low coefficient of friction tribofilm at the sliding interlayer. However, after this stage, the coefficient of friction begins to rise and does not maintain its null value. From the results, this may be a consequence of several factors. Firstly, the lowest Young’s modulus of this coating yields a high strain of the coating, which is evident in the steel ball (Figure 10d), resulting not only in the broader wear track but also on the smearing of the interface tribolfilm to the edges of the wear track. Therefore, the protective effect of the interface tribofilm disappears. Moreover, as stated before, the Cu-C100 has a more graphitic like behavior, which implies that the absence of humidity results in weaker tribological properties, as discussed earlier [54]. These factors contribute for the maintenance of the disordered structure and, contrary to the thinner coatings, does not contribute to a null friction coefficient.

## Figures and Tables

**Table 1:** Designation, deposition parameters, and thickness of copper and carbon deposited thin films.

Designation	Deposition Parameters			Thickness (nm)
	D_dep_ Cu (W·mm^−2^)	D_dep_ C (W·mm^−2^)	Time (s)	
Cu	3.2 × 10^−2^	0	600	600
C15	0	2.5 × 10^−2^	300	15
C60	0	2.5 × 10^−2^	1800	60
C100	0	2.5 × 10^−2^	3600	100

**Table 2:** X-ray photoelectron spectroscopy (XPS) data from the deconvolution of C1s high-resolution spectra of carbon coatings.

C Bonds		Surface		
		C15	C60	C100
sp^1^	Peak Position (eV)	282.6	282.4	n.d.
	Concentration (%)	35.7	5.2	n.d.
	FWHM (eV)	1.4	1.9	n.d.
sp^2^	Peak Position (eV)	284.1	284.2	284.2
	Concentration (%)	57.2	60.3	47.4
	FWHM (eV)	1.9	1.3	1.3
sp^3^	Peak Position (eV)	n.d.	284.9	285.0
	Concentration (%)	n.d.	18.0	34.0
	FWHM (eV)	n.d.	1.7	1.4
C–O	Peak Position (eV)	286.1	286.3	286.2
	Concentration (%)	7.1	9.3	15.2
	FWHM (eV)	2.0	1.7	2.2
O–C–O	Peak Position (eV)	287.6	287.5	n.d.
	Concentration (%)	3.4	7.2	n.d.
	FWHM (eV)	2.0	1.7	n.d.

n.d.—not determined by the fitting process.

**Table 3:** Relative intensity ratio of Raman peaks and G peak position of carbon thin films.

Surface	G Peak Position (cm^−1^)	Intensity Ratios		
		*I_D_/I_G_*	*I_C_/I_G_*	*I_D_/I_D’_*
C15	1574	0.57	0.30	3.0
C60	1551	0.62	0.25	3.3
C100	1531	0.37	0.04	5.2

**Table 4:** Hardness (*H*) and Young’s modulus (*E*) of the deposited surfaces and bulk copper.

Surface	*H* (GPa)	*E* (GPa)
Cu	2.64 ± 0.07	168 ± 10
C15	2.75 ± 0.26	164 ± 13
C60	2.86 ± 0.07	126 ± 5
C100	2.95 ± 0.10	101 ± 2
Bulk Copper	1.91 ± 0.09	144 ± 19
Bulk Copper (1 N)	0.99 ± 0.02	-

## References

[1] Pellicer E., Varea A., Pané S., Nelson B.J., Menéndez E., Estrader M., Suriñach S., Baró M.B., Nogués J., Sort J. (2010). Nanocrystalline electroplated Cu–Ni: Metallic thin films with enhanced mechanical properties and tunable magnetic behavior. Adv. Funct. Mater..

[2] Hsu K.S., Lin M.T., Tong C.J. (2011). The measurement of cyclic creep behavior in copper thin film using microtensile testing. MEMS and Nanotechnology.

[3] Torazawa N., Hirao S., Kanayama S., Korogib H., Matsumoto S. (2016). The development of Cu filling and reliability performance with Ru-Ta alloy barrier for Cu interconnects. J. Electrochem. Soc..

[4] Gambino J., Chen F., He J. Copper interconnect technology for the 32 nm node and beyond. Proceedings of the IEEE Custom Integrated Circuits Conference.

[5] Josell D., Brongersma S.H., Tőkei Z. (2009). Size-dependent resistivity in nanoscale interconnects. Annu. Rev. Mater. Res..

[6] Lazarus N., Meyer C.D., Bedair S.S., Song X., Boteler L.M., Kierzewski I.M. (2013). Thick film oxidation of copper in an electroplated MEMS process. J. Micromech. Microeng..

[7] Chen H., Filleter T. (2015). Effect of structure on the tribology of ultrathin graphene and graphene oxide films. Nanotechnology.

[8] Savage N. (2012). Materials science: Super carbon. Nature.

[9] Kim H.-J., Kim D.-E. (2015). Water lubrication of stainless steel using reduced graphene oxide coating. Sci. Rep..

[10] Berman D., Erdemir A., Sumant A.V. (2014). Graphene: A new emerging lubricant. Mater. Today.

[11] Khan Z.H., Kermany A.R., Öchsner A., Iacopi F. (2017). Mechanical and electromechanical properties of graphene and their potential applications in MEMS. J. Phys. D: Appl. Phys..

[12] Yuanhong X., Jingquan L. (2016). Graphene as transparent electrodes: Fabrication and new emerging applications. Small.

[13] Kim K.-S., Lee H.-J., Lee C., Lee S.-K., Jang H., Ahn J.-H., Kim J.-H., Lee H.-J. (2011). Chemical vapor deposition-grown graphene: The thinnest solid lubricant. ACS Nano.

[14] Lee C., Wei X., Li Q., Carpick R., Kysar J.W., Hone J. (2009). Elastic and frictional properties of graphene. Phys. Status Solidi B.

[15] Ko J.-H., Kwon S., Byun I.-S., Choi J.S., Park B.H., Kim Y.-H., Park J.Y. (2013). Nanotribological properties of fluorinated, hydrogenated, and oxidized graphenes. Tribol. Lett..

[16] Li Q., Lee C., Carpick R.W., Hone J. (2010). Substrate effect on thickness-dependent friction on graphene. Phys. Status Solidi B.

[17] Wenjuan Z., Low T., Perebeinos V., Bol A.A., Zhu Y., Yan H., Tersof J., Avouris P. (2012). Structure and electronic transport in graphene wrinkles. Nano Lett..

[18] Marchena M., Wagner F., Arliguie T., Zhu B., Johnson B., Fernández M., Chen T.L., Chang T., Lee R., Pruneri V. (2018). Dry transfer of graphene to dielectrics and flexible substrates using polyimide as a transparent and stable intermediate layer. 2D Mater..

[19] Banhart F., Kotakoski J., Krasheninnikov A.V. (2011). Structural defects in graphene. ACS Nano.

[20] Parveen K., Wani M.F. (2017). Synthesis and tribological properties of graphene: A review. Jurnal Tribologi.

[21] Peng Y., Wang Z., Zou K. (2015). Friction and wear properties of different types of graphene nanosheets as effective solid lubricants. Langmuir.

[22] Kinoshita H., Kume I., Tagawa M., Ohmae N. (2004). High friction of a vertically aligned carbon-nanotube film in microtribology. Appl. Phys. Lett..

[23] Berman D., Deshmukh S.A., Sankaranarayanan S.K.R.S., Erdemir A., Sumant A.V. (2014). Extraordinary macroscale wear resistance of one atom thick graphene layer. Adv. Funct. Mater..

[24] Strmčnik E., Majdič F., Kalin M. (2019). Water-lubricated behaviour of AISI 440C stainless steel and a DLC coating for an orbital hydraulic motor application. Tribol. Int..

[25] Su F., Chen G., Sun J. (2019). Synthesis of hydrogenated DLC film by PECVD and its tribocorrosion behaviors under the lubricating condition of graphene oxide dispersed in water. Tribol. Int..

[26] Tyagi A., Walia R.S., Murtaza Q., Pandey S.M., Tyagi P.K., Bajaj B. (2019). A critical review of diamond like carbon coating for wear resistance applications. Int. J. Refract. Met. Hard Mater..

[27] Nißen S., Heeg J., Wienecke M., Behrend D., Warkentin M. (2018). Enhancing adhesion strength of a-C:H:Cu composite coatings on Ti6Al4V by graded copper deposition in a rf PVD/PECVD hybrid process. Surf. Coat. Technol..

[28] Kang K.-N., Lee J., Lee K.Y., Kang Y., Park Y.S. (2019). Tribological and electrical properties of chromium-doped carbon films fabricated by unbalanced magnetron sputtering for medical stents. J. Nanosci. Nanotechnol..

[29] Chen Z., He H., Xiao C., Kim S.H. (2018). Effect of humidity on friction and wear—A critical review. Lubricants.

[30] Pinho A.C., Piedade A.P. (2013). Zeta potential, contact angles, and AFM imaging of protein conformation adsorbed on hybrid nanocomposite surfaces. ACS Appl. Mater. Interfaces.

[31] Piedade A.P., Nunes J., Vieira M.T. (2008). Thin films with chemically graded functionality based on fluorine polymers and stainless steel. Acta Biomat..

[32] Piedade A.P., Vieira M.T., Martins A., Silva F. (2008). In vitro behaviour of nanocrystalline silver-sputtered thin films. Nanotechnology.

[33] Carvalho D., Sousa T., Morais P.V., Piedade A.P. (2016). Polymer/metal nanocomposite coating with antimicrobial activity against hospital isolated pathogen. Appl. Surf. Sci..

[34] Reichelt K., Jiang X. (1990). The preparation of thin films by physical vapour depositions methods. Thin Solid Films.

[35] Greene J.E. (2017). Tracing the recorded history of thin-film sputter deposition: From the 1800s to 2017. J. Vac. Sci. Technol. A.

[36] Martins L.G.P., Song Y., Zeng T., Dresselhaus M.S., Kong J., Araujo P.T. (2013). Direct transfer of graphene onto flexible substrates. PNAS.

[37] Lide D.R. (2004). Handbook of Chemistry and Physics.

[38] Danno T., Okada Y., Kawaguchi J. (2004). XPS study of carbyne-like carbon films. AIP Conf. Proceed..

[39] Ferrari A.C., Robertson J. (2000). Interpretation of Raman spectra of disordered and amorphous carbon. Phys. Rev. B.

[40] Hong J., Park M.K., Lee E.J., Lee D., Hwang D.S., Ryu S. (2013). Origin of new broad Raman D and G peaks in annealed graphene. Sci. Rep..

[41] Casari C.S., Milani A. (2018). Carbyne: From the elusive allotrope to stable carbon atom wires. MRS Commun..

[42] Zabel J., Nair R.R., Ott A., Georgiou T., Geim A.K., Novoselov K.S., Casiraghi C. (2012). Raman spectroscopy of graphene and bilayer under biaxial strain: Bubbles and balloons. Nano Lett..

[43] Rafiee J. (2012). Wetting transparency of graphene. Nat. Mat..

[44] Palaniselvam T., Aiyappa H.B., Kurungot S. (2012). An efficient oxygen reduction electrocatalyst from graphene by simultaneously generating pores and nitrogen doped active sites. J. Mater. Chem..

[45] Lv Q., Si W., He J., Sun L., Zhang C., Wang N., Yang Z., Li X., Wang X., Deng W. (2018). Selectively nitrogen-doped carbon materials as superior metal-free catalysts for oxygen reduction. Nat. Commun..

[46] Xue L., Li Y., Liu X., Liu Q., Shang J., Duan H., Dai L., Shui J. (2018). Zigzag carbon as efficient and stable oxygen reduction electrocatalyst for proton exchange membrane fuel cells. Nat. Commun..

[47] Eckmann A., Felten A., Mishchenko A., Britnell L., Krupke R., Novoselov K., Casiraghi C. (2012). Probing the nature of defects in graphene by Raman spectroscopy. Nano Lett..

[48] Lu L., Tao N.R., Wang L.B., Ding B.Z., Lu K. (2001). Grain growth and strain release in nanocrystalline copper. J. Appl. Phys..

[49] Vaz D., Piedade A.P. (2018). Structure and mechanical properties of a copper combustion chamber throughout its life cycle. Metals.

[50] Zhang S. (2010). Nanostructured Thin Films and Coatings: Mechanical Properties.

[51] Kotrechko S., Mikhailovskij I., Mazilova T., Sadanov E., Timoshevskii A., Stetsenko N., Matviychuk Y. (2015). Mechanical properties of carbine: Experiment and simulations. Nanoscale Res. Lett..

[52] Mirzaeifar R., Qin Z., Buehler M.J. (2014). Tensile strength of carbine chains in varied chemical environments and structural length. Nanotechnology.

[53] Timoshevskii A., Kotrechko S., Matviychuk Y. (2015). Atomic structure and mechanical properties of carbyne. Phys. Rev. B.

[54] Bryant P.J., Gutshall P.L., Taylor L.H. (1964). A Study of mechanisms of graphitic friction and wear. Wear.

